# Decreasing acid value of fatty acid ethyl ester products using complex enzymes

**DOI:** 10.3389/fbioe.2024.1355009

**Published:** 2024-02-08

**Authors:** Yuting Li, Jingjing Guo, Shangde Sun

**Affiliations:** Lipid Technology and Engineering, Henan University of Technology, Zhengzhou, China

**Keywords:** fatty acid ethyl ester, Semen Abutili seed oil, acid value, complex enzymes, transesterification, esterification

## Abstract

Recently, enzymatic method has been used to prepare biodiesel using various oils. But the high acid value of the biodiesel product using enzyme as a catalyst has been one issue. In this work, an attempt to reduce the acid value of fatty acid ethyl ester (FAEE) product to satisfy the specified requirement (AV ≤ 0.5 mgKOH/g), a complex enzyme-catalyzed method was used for the ethanolysis of Semen Abutili seed oil (SASO) (AV = 5.5 ± 0.3 mgKOH/g). The effects of various variables (constituents of complex enzyme, type and addition of water removal agent, time, temperature, enzyme addition load, substrate ratio) on the enzymatic reaction were investigated. The optimal reaction conditions were: 1% addition of liquid lipase Eversa^®^ Transform 2.0% and 0.8% of enzyme dry powder CALB, reaction temperature 35°C, alcohol-oil ratio 9:1 (mol/mol), 0.8 g/g of 4A-MS and reaction time 24 h. Under the optimal reaction conditions, the FAEE yield was 90.8% ± 1.5% and its acid value was decreased from 12.0 ± 0.2 mgKOH/g to 0.39 ± 0.10 mgKOH/g. In further evaluating the feasibility of preparing FAEE from SASO, the FAEE products obtained under the optimal reaction conditions were purified and evaluated with reference to the ASTM D6751 standard for the main physicochemical indexes. The results obtained were in accordance with the requirements except for the oxidative stability.

## 1 Introduction

The global demand for biodiesel, a renewable and sustainable alternative to fossil fuels, has a significant growth in recent years (Thangaraj et al., 2018). This surge in interest can be attributed to the future trends for reducing greenhouse gas emissions and mitigating the environmental impact of traditional fossil fuels (Murugesan et al., 2009). In response to this demand, extensive research efforts have been focused on the development of the efficient and environmentally friendly biodiesel production. Sources of feedstock for biodiesel were mainly edible oils, animal fats and waste cooking oils (Zulqarnain et al., 2021; Kharia et al., 2023). However, research on its application has been limited due to its high cost, shortage of resources or high free fatty acid and water contents.

Fatty acid ethyl ester (FAEE) is considered as a sustainable and environmentally friendly raw material of biodiesel (Gutiérrez et al., 2009; Pisarello et al., 2010; Jiang et al., 2013; Fadhil and Abdulahad, 2014; Zanuttini et al., 2014). The use of ethanol (a renewable alcohol) enables biodiesel to be 100% renewable compared to the use of methanol as a feedstock for transesterification (Lapuerta et al., 2008). Alternatively, the use of ethanol as an acyl receptor can also reduce the methanol disinhibition effects (Verma and Sharma, 2016).

FAEE can be obtained through the esterification of free fatty acid (FFA) and the ethanolysis of triglyceride (TG) from various feedstock. However, there are some issues of the former method, including a complex process, high energy consumption, and large amount of waste water (Ahmed and Huddersman, 2022). Conversely, due to the advantages of low corrosiveness, easy separation of products and high yield, ethanolysis has been one of the most commonly used method for the production of FAEE (Bhatia, 2014). Based on the type and presence of catalysts, ethanolysis can be classified into chemical catalysis, supercritical fluid catalysis, and enzymatic catalysis. Additionally, chemical catalysis can be further divided into acid-catalyzed and base-catalyzed methods.

Marchetti et al. (Marchetti et al., 2008) investigated the sulfuric acid-catalyzed preparation of FAEE from oils with high fatty acid content, which resulted in 95% conversion under the optimal process. However, the application of acidic catalyst normally required high temperature and pressure. In addition, the acid catalyst has strict requirements for the moisture in oil (<0.5%), as excessive water will reduce the product yield and lead to the deactivation of the acid catalyst (Hu et al., 1993; Aafaqi et al., 2004).

Alkali catalysts, such as KOH and NaOMe, are the most commonly used catalysts for the preparation of FAEE in industry. However, the application of alkali catalysts typically requires feedstocks with low contents of free fatty acid (<0.5%) and moisture (<0.3%) to avoid saponification during the reaction process, which can result in a cumbersome washing process (Sánchez et al., 2000; Cho et al., 2012; Lv et al., 2017). Additionally, this method generally produces a high volume of wastewater (0.2 tons/ton of biodiesel), which is detrimental to environmental protection and energy conservation.

Compared with the traditional preparation method of FAEE, the supercritical method has several advantages, such as low raw material requirements, easy separation of products, and environmentally friendly. However, this method typically requires demanding reaction conditions: temperature >200 °C, pressure >15 MPa, and alcohol-oil ratio >20:1 (mol/mol) (Silva et al., 2011). Which results in high production costs.

Enzymatic synthesis of biodiesel, as a promising alternative to traditional chemical methods, has gained considerable attention due to its excellent advantages (Szczęsna Antczak et al., 2009). Statistics reveal a steady increase in the number of research studies focusing on enzymatic biodiesel synthesis over the past decades (Supplementary Figure S1). This trend underscores the growing significance of enzymatic approaches in the biodiesel production landscape. Enzymatic biodiesel synthesis offers several compelling advantages over chemical methods (Fjerbaek et al., 2009). Firstly, it provides access to a wide range of raw materials, including non-edible and waste feedstocks, thereby reducing competition with food production (Demirbas et al., 2016; Abdul Hakim Shaah et al., 2021). Secondly, enzymatic processes are inherently more environmentally friendly, as they typically operate under mild reaction conditions, leading to reduced energy consumption and the formation of undesirable byproducts and environmental pollutants (Mangas-Sánchez and Adlercreutz, 2015; Arumugam and Ponnusami, 2017). Moreover, enzymatic biodiesel production is characterized by more efficient waste disposal strategies, making it as a viable and sustainable option for the future of biofuels (Toldrá-Reig et al., 2020).

During the production of biodiesel through single-enzymatic synthesis, the high acid value of feedstocks has been a critical issue (Singh et al., 2018). High acid value of feedstocks can result in some undesirable consequences, such as a decrease in catalytic performance of enzymes and quality of biodiesel (Yeşilyurt et al., 2019; Jafarihaghighi et al., 2020). Therefore, addressing the issue of high acid value is paramount to ensure the successful adoption of enzymatic biodiesel synthesis (Ateeq et al., 2022). Despite the typical challenges associated with enzyme applications, such as long reaction times, high costs, and separation difficulties, the advantages of complex enzyme catalysts outweigh their disadvantages. This ultimately leads to improved production efficiency of biodiesel when using the feedstock with high fatty acid content.

Abutilon theophrasti Medicus is widely distributed throughout China, both in the wild and in cultivation. Abutilon has an oil content of about 20%. Semen Abutili seed oil (SASO) contains high levels of sixteen and eighteen carbon chain unsaturated fatty acids, which can be used as a good raw material for biodiesel preparation (Sun et al., 2021). However, the virgin SASO had an acid value of 5.5 ± 0.3 mgKOH/g. Therefore, it is necessary to reduce the acid value of its fatty acid ethyl ester product. To solve this issue, a combination of enzymes was employed in this work and the effects of enzyme ratios and reaction conditions were investigated. This work can provide valuable insight for the challenge of reducing the high acid value of biodiesel using enzymatic method in the biofuel industry.

## 2 Materials and methods

### 2.1 Materials

SASO was prepared in our laboratory and obtained by solvent extraction method with an acid value of 5.5 ± 0.3 mgKOH/g. All the commercial lipases (lipase A from *Candida sp*. (CALA), Eversa^®^ Transform 2.0, and lipase B from *Candida sp*. (CALB) were acquired from Novozymes A/S (Denmark). The ethanol was supplied from Kemiou Chemical Reagent Co., Ltd (Tianjin, China). Molecular sieves 4A (4A-MS) was purchased from Aladdin Biochemical Technology Co., Ltd. (Shanghai, China). Blue silica gel was acquired from Komeo Chemical Reagent Co., Ltd. (Tianjin, China). Acrylic acid super absorbent polymer (SAP) was bought from Wanhua Chemical Group Co., Ltd. (Yantai, China). Dialysis Bag MD 44 was obtained from Sobalai Technology Co., Ltd. (Beijing, China).

### 2.2 The method of complex lipases removing free fatty acid

Under the conditions of enzyme addition of 4% liquid lipase (w/w, SASO), 0.8% enzyme dry powder (w/w, SASO), 7:1 (mol/mol) substrate ratio of alcohol to oil, 0.6 g/g of dehydrating agent, 35°C, the reaction was carried out in a 25 mL round bottom flask and then placed in a constant temperature water bath oscillator. Samples were taken out at regular intervals for centrifugation in a centrifuge tube, and a certain amount of the upper sample was used for GC and acid value analysis.

### 2.3 Analysis methods

#### 2.3.1 Determination of AV

Acid value is determined according to AOCS Official Method Cd 3d-63.

#### 2.3.2 Determination of the composition of the FAEE product

High-temperature GC was used to analyze the FAEE yield and other components in the reaction system.

The samples were dissolved in hexane, de-watered and filtered through a membrane before Agilent 7890B high temperature GC analysis. Component separation was performed using a chromatographic column JW122-1131DB-1ht (30 m × 250 μm×0.10 μm) with a flame ionization detector. Nitrogen was used as carrier gas at a flow rate of 4 mL/min. The inlet temperature was 280°C and the detector temperature was 350°C. The column temperature was initially set at 160°C, and increased to 220°C at a rate of 60 °C/min and retained at 220°C for 3 min, then increased to 290°C at a rate of 20 °C/min, and then increased to 320°C by 6 °C/min and hold at 320°C for 1 min, and finally elevated to 360°C by 20 °C/min and hold at 360°C for 7 min.

The contents of each component were quantified by external standard method and area normalization method.

#### 2.3.3 Establishment of standard curve

The standard curve for the substances is shown in Supplementary Figure S2.

#### 2.3.4 Calculation of FAEE content and concentration of each component

FAEE yield was calculated as follows (Sánek et al., 2013; Huang Z et al., 2015; Guo et al., 2020):

$$FAEE\;yield\left(\%\right)=\frac{A_{FAEE}}{A_{MG}+A_{DG}+A_{TG}+A_{FFA}+A_{FAEE}}\times 100\%$$

A is the chromatogram area of the product. The concentration of each component can be calculated from the standard curve.

### 2.4 Purification of SASO fatty acid ethyl esters

The purification of the synthesized FAEE product of SASO was carried out using molecular distillation under the following conditions: heating temperature of 120°C, scraping speed of 50 rpm, injection volume of 2 mL/min, and pressure of 50 Pa. The light phase product was obtained as the FAEE product.

### 2.5 Physico-chemical indexes determination of FAEE

The FAEE product of SASO were tested for acid value, moisture content, iodine value, oxidative stability, cetane number, and other indexes in accordance with ASTM standards.

### 2.6 Statistical analysis

All experiments were performed at least in triplicate. Results were expressed as average ±S.E.M. SPSS 20.0 was employed to analyze the data. Statistical significance was considered at *p* < 0.05.

## 3 Results and discussion

### 3.1 Lipase screening

Lipase contains an amphiphilic amino acid chain, the lid, which is responsible for the activation of the enzyme due to its mobility and protection of the enzyme’s active site (Khan et al., 2017). Differences in the lid structure, active center structure, and the amount of water needed to activate lipase resulted in different specificities and catalytic activities (Secundo et al., 2006; Tongboriboon et al., 2010).

What’s a side note, the catalytic activity of the liquid lipase was greater than that of the corresponding enzyme dry powder, probably because the right amount of water facilitates the opening of the lid structure covered by the active site of lipase, and maintains the stability of the lipase structure and provides a useable oil-water interface (Huang Z et al., 2015).

From Supplementary Figure S3, it can be seen that FAEE product has the highest yield when catalyzed by the liquid lipase Eversa^®^ Transform 2.0. This was due to the fact that the lipase Eversa^®^ Transform 2.0 has a smaller lid structure, which allows for easy opening of the lid at the interfaces of oil and water so that shows a higher catalytic activity. However, the lipase CALB has a lid structure of 5 amino acids and CALA has 9–10 amino acids. (Uppenberg et al., 1994; Ericsson et al., 2008). In addition, the “channel-like” stucture of the lipase Eversa^®^ Transform 2.0 made binding of SASO to ethanol and release of the product easier, which resulted in a faster reaction rate than the lipases CALA and CALB. It also suggests that liquid lipase has better tolerance to ethanol (Li et al., 2013).

For maximum FAEE yield, the lipase should be able to convert all glycerides (including mono-, di-, and triglycerides) as well as free fatty acids into FAEE. The result showed that the lowest acid value of FAEE (0.2 ± 0.12 mgKOH/g) was found in the final system of the reaction using dry enzyme powder of CALB as a catalyst. This may be due to the absence of interfacial activation of lipase CALB (Tongboriboon et al., 2010) or the low water requirement to activate lipase CALB (Martinelle et al., 1995). The main reason is attributed to the higher catalytic esterification capacity of the dry enzyme powder of CALB.

As a consequence, the reaction requires both a favorable liquid enzyme with the ability to catalyze esterification to maintain a high yield, and a certain amount of enzyme dry powder capable of esterification with free fatty acids to reduce the acid value to further maximize the yield. (Christopher et al., 2014). Therefore, a combination of enzymes (liquid lipase Eversa^®^ Transform 2.0 and enzyme dry powder of CALB) was used for the next studies.

### 3.2 Comparation of different removal methods of water

As can be seen from the experimental results (Supplementary Figure S4), preparation of liquid lipase Eversa^®^ Transform 2.0 catalyzed under different dehydrating agent conditions, the FAEE yield was: 4A-MS (86.3% ± 1.2%) > blue silica (83.1% ± 1.5%) > SAP (76.2% ± 1.0%), and the acid value of FAEE product was: SAP (4.5 ± 0.2 mgKOH/g) > blue silica gel (4.4 ± 0.1 mgKOH/g) > 4A-MS (3.2 ± 0.1 mgKOH/g). Three water removals have similar FAEE yield, but 4A-MS is able to achieve a lower acid value while maintaining a high FAEE yield.

During the enzymatic synthesis of FAEE, a relatively small amount of water was required in organic solvent to maintain enzyme activity (Atadashi et al., 2012). It was demonstrated that for every 1% of fatty acids in the raw material, 650 mg/kg of water was produced by the esterification reaction (Liu et al., 2013). Due to the high content of free fatty acids in raw material used to synthesize FAEE, a large amount of water could be generated during the esterification reaction with ethanol. However, excess water can facilitate hydrolysis of the oil. Furthermore, water in the reaction system may inhibit the positive progression of the lipase-catalyzed reaction and result in the high acid value of the product (Hájek et al., 2012; Nguyen et al., 2018). During the reaction, the surface of the lipase Eversa^®^ Transform 2.0 requires a layer of essential water to maintain a certain active conformation for its catalytic activity (Molina-Gutiérrez et al., 2021). Therefore, the dehydration capacity of the water removal agent should be appropriate, so that not only the acid value of the FAEE product is desired, but also an essential layer of water is not taken away from the surface of the enzyme.

Difference ability of dehydrating agents in water removal can be explained based on the principle of removing water and the different specific surface areas of the materials. Firstly, SAP is a white powder with small particle size (0.2–0.9 mm), large specific surface area, and many grooves on the surface, which provides a large space for SAP to store water (Gu et al., 2015). In addition, SAP has a strong water removal capacity and it can efficiently take away the essential water from the surface of the lipase Eversa^®^ Transform 2.0 molecule, thus putting the lipase in a rigid inactivation state and reducing the catalytic activity. These resulted in the lowest FAEE yield and the highest acid value when the reaction was catalyzed with the lipase Eversa^®^ Transform 2.0. Secondly, compared with other water removing agents (4A-MS, SAP), blue silica gel has a smaller specific surface area and weaker adsorption surface polarity. In addition, the pore size of blue silica gel is larger than the molecular size of ethanol, and the adsorption of ethanol by silica gel may negatively affect the reaction and thus getting a poorer result (Wang et al., 2006). Finally, as the specific surface area of 4A-MS is similar to that of blue silica gel, but the polar effect of the adsorption surface is stronger and its water removal effect was better than blue silica gel. Therefore, 4A-MS was the best choice of water removal agent for this work. In contrast to other dehydrating agents, the lipase Eversa^®^ Transform 2.0 in the 4A-MS system had higher catalytic activity and lower acid value of the FAEE product, indicating that 4A-MS did not take away essential water from the enzyme surface. For consequence, 4A-MS was used for the next research.

### 3.3 Effect of the proportion of complex enzymes

In the screening of enzyme types, we chose Eversa^®^ Transform 2.0, a low-cost liquid enzyme with good catalytic effeciency, to be compounded with the dry powder enzyme CALB. The function of CALB is to catalyze the esterification of free fatty acids with ethanol to obtain more product that reduces the acid value. Also, Eversa®Transform 2.0 is effective in increasing yield due to its non-specificity (Christopher et al., 2014) and better tolerance to ethanol and phospholipids (Li et al., 2013). As shown in Supplementary Figure S5, at a certain enzyme dry powder CALB (0.8%) addition, with the increasing addition load of liquid lipase Eversa^®^ Transform 2.0 from 1% to 8%, there was an increase in FAEE yield (from 91.2% ± 1.2% to 97.6% ± 1.5%). However, a substantial rise in the acid value of the product was observed (from 2.7 ± 0.1 mgKOH/g to 6.2 ± 0.1 mgKOH/g). The reason is that the volume of enzyme added not only affected the reaction rate, but also imported more water into the reaction system, which in turn affected the acid value of FAEE product.

The relationship between the acid value of FAEE and the moisture content of the system is displayed in Supplementary Figure S6. In fact, the proportion of the complex enzyme not only affects the rate of the reaction, at the same time, since the liquid lipase itself contains water, its addition increases the moisture content of the system which in turn affects the acid value of the FAEE product. As can be deduced from the linear relationship between moisture and acid value in the system that the acid value of biodiesel catalyzed by lipase could meet the requirement (AV≤0.5 mgKOH/g) only when the moisture content in the system was no more than 500 mg/kg. In order to achieve the required acid value of the FAEE product (≤0.5 mgKOH/g), a combined complex enzyme with 1% liquid lipase Eversa^®^ Transform 2.0% and 0.8% enzyme dry powder CALB were the best choice.

### 3.4 Effect of 4A-MS addition load

With the increase of 4A-MS addition load, the acid value of FAEE product was gradually decreased. But the yield of FAEE was also slowly declined (Supplementary Figure S7). This may be due to the fact that the removal of water from the reaction system is enhanced by the increasing addition load of 4A-MS, which in turn promotes the positive direction of the esterification. However, the increase in 4A-MS addition load limited enzyme diffusion, increased resistance to migration of the reaction, impeded access of substrates (SASO and ethanol) to the complex enzymes active sites, and retarded the response velocity (Nguyen et al., 2018). In addition, the increasing addition load of 4A-MS would take away the essential water from the lipase surface layer, which leaves the enzyme molecule in a rigid deactivated state and in turn weakens the catalytic activity of the enzyme and slows down the reaction rate.

Accordingly, to reduce the acid value of the FAEE product below the required standard (≤0.5 mgKOH/g) and hold high FAEE yield, 0.8 g/g 4A-MS was added to carry out the following experiments.

### 3.5 Effect of substrate ratio

When the alcohol-oil substrate is added from 3:1 (mol/mol) to 9:1 (mol/mol), the FAEE yield was sharply increased from 33.3% ± 1.0% to 90.8% ± 1.5%, while the acid value of product was dramatically decreased from 3.8 ± 0.1 mgKOH/g to 0.36 ± 0.06 mgKOH/g (Supplementary Figure S8). This is owing to the fact that an increase in the alcohol-oil substrate ratio can promote a shift in the reaction equilibrium toward more FAEE production of SASO. With a continued increase in the substrate ratio (>9:1), the yield of FAEE was decreased, while the acid value of the product was also increased. This may be due to the reason that an increase in ethanol concentration in the reaction system affects the folding structure of lipase protein (Eversa^®^ Transform 2.0 and CALB), which leads to a decrease in the catalysing ability of lipase (Liu et al., 2013). Alternatively, there is also a reason that high alcohol concentration weakens the activity of the enzyme by stripping the essential water molecules and unfavorably unfolding it into a more helical state (Lotti et al., 2015). At the same time, the increase of ethanol addition also increased the water content in the reaction system, which inhibited the positive transesterification reaction and resulted in an increase of acid value. In addition, high alcohol-oil ratio can affect the separation of products. In the meantime, the additional ethanol can make the mass concentration of SASO in the system smaller, resulting in a slow formation rate of enzyme-substrate complex.

This demonstrated that an alcohol-oil ratio of 9:1 (mol/mol) was the appropriate substrate ratio for the complex enzyme catalyzed the preparation of FAEE product of SASO, which was greater than the alcohol-oil molar ratio for the immobilized lipase (from *Pseudomonas cepacia*) catalyzed ethanolysis of Jatropha curcas hair oil (4:1) (Aransiola, 2013). The results also showed that the combination of the complex enzymes had high alcohol resistance and could not easily be inactivated in high ethanol environments.

### 3.6 Effect of reaction temperature

Reaction temperature is an important parameter affecting the catalytic activity of lipase and reaction rate. The yield of FAEE was increased to a maximum value of 90.83% ± 1.5% when the temperature was varied up from 25°C to 35°C, and the acid value of FAEE product was decreased to 0.36 ± 0.06 mgKOH/g (Supplementary Figure S9). This may be due to the fact that higher temperatures foster the collision of lipase and substrate as well as the formulation of enzyme-substrate composite, thus increasing the efficiency of the reaction. In addition, elevating temperature also reduces the viscosity of reaction system, thereby alleviating mass transfer limitations and facilitating the effective diffusion of the substrates (Guo et al., 2020). It was observed, however, that there was a significant decrease in FAEE yield when the temperature was increased from 35°C to 65 °C. This may be due to the influence of higher temperature (>35°C) on the structure of lipase, resulting in the inactivation of lipase.

Therefore, the reaction temperature of 35°C is the optimum temperature for the preparation of FAEE catalyzed by the complex enzymes. Compared with the preparation of FAEE by ethanolysis of murumuru oil using the complex enzymes, Lipase AK (from *Pseudomonas fluorescens*) and Lipase PS (from *P. cepacia*) (the optimum temperature of 45°C), the reaction temperature (35°C) in our work was lower and less energy consuming (Tongboriboon et al., 2010).

### 3.7 Changes in the content of each component during the preparation of FAEE catalyzed by complex enzymes

SASO mainly consists of TG and FFA, and two parallel reactions simultaneously exist in this reaction system: i) ester exchange reaction (TG is reacted with ethanol to form MG, DG and FAEE), and ii) esterification reaction (FFA is reacted with ethanol to form FAEE). It can be observed from Supplementary Figure S10 that the yield of FAEE was rapidly increased to 65.2% ± 1.3% during the first 6 h of the reaction. This was followed by a gradual increase to 89.2% ± 1.8%. When reaction time was further increased to longer than 24 h, the yield of FAEE was remained almost constant (∼90%). The equilibrium time of this reaction (24 h) was similar with that of the lipase *Rhizomucor miehei* catalyzed preparation of FAEE from microalgae oil (24 h) (Huang J et al., 2015).

In the first 6 h of the reaction, the content of TG was rapidly decreased and the content of MG was gradually increased. At the same time, the content of DG was gradually increased during the first 3 h of the reaction. However, when reaction time was >6 h, the contents of TG, DG and MG were all gradually reduced. This indicated that the transesterification reaction of TG with ethanol was the main reaction in the first 6 h. However, when the reaction time was longer than 6 h, the main transesterification reactions of DG and MG with ethanol were observed. At the same time, the FFA content was gradually dropped throughout the reaction, and to less than 0.25% at 12 h. These suggested that the esterification reaction also existed simultaneously in this reaction system, but was not the main reaction.

### 3.8 FAEE products

The physical and chemical indexes (except oxidative stability) of the SASO FAEE product were in compliance with the requirements of ASTM D6751 (as shown in Supplementary Table S1). To address the issue of oxidative stability of FAEE product of SASO, antioxidants such as butylated hydroxyanisole (BHA) and butylated hydroxytoluene (BHT) were added in the subsequent study. The addition of antioxidants both improves the oxidative stabilities of biodiesel product and decreases the emission of nitrogen oxides (Ryu, 2010). Biodiesel prepared from SASO has a lower viscosity (4.54 mm^2^/s) than that from rapeseed oil (4.83 mm^2^/s) and palm oil (5.70 mm^2^/s), which was more conducive to the flow of biodiesel in engines (Ryu, 2010). The density of the FAEE product prepared with SASO was 0.870 g/cm^3^, which is lower than the 0.880 g/cm^3^ prepared from sunflower oil, as well as 0.882 g/cm^3^ for rapeseed oil and 0.899 g/cm^3^ for castor oil in other studies (Karmakar et al., 2010). The SASO FAEE product has a flash point of 174°C, which is qualified by ASTM D6751. These suggested that the biodiesel prepared by SASO had relatively excellent injection and atomization properties, which can be used as a potential renewable oil resource for biodiesel preparation.

## 4 Conclusion

In this study, an innovative method using a complex enzyme to prepare FAEE product with the low acid value was successfully developed. The optimal reaction conditions were 1% liquid lipase Eversa^®^ Transform 2.0% and 0.8% enzyme dry powder CALB, reaction temperature 35°C, alcohol-oil ratio 9:1 (mol/mol), 4A-MS addition load 0.8 g/g and reaction time 24 h. Under these conditions, the FAEE yield of the product was 90.8% ± 1.5% and its acid value was 0.39 ± 0.10 mgKOH/g. The physicochemical indicators of the purified FAEE product were analyzed according to ASTM D6751 standards, and the FAEE product from SASO were all in accordance with the standards except for the oxidative stability. The oxidative stability of the FAEE product can be improved by adding antioxidants.

This research represents a crucial step towards improving the enzymatic synthesis of SASO into low acid value FAEE product. It also provides a new idea for feedstock selection for biodiesel using SASO. In summary, the transition from chemical to enzymatic methods for biodiesel synthesis, coupled with a focus on reducing acid value, suggests a promising future for the green and sustainable production of biodiesel. This research aims to address critical research gaps and contribute to the broader understanding of enzymatic biodiesel synthesis while meeting international standards of biodiesel.

## Data Availability

The datasets presented in this study can be found in online repositories. The names of the repository/repositories and accession number(s) can be found in the article/Supplementary Material.
