# Supplementary material for: Decreasing acid value of fatty acid ethyl ester products using complex enzymes
Source: Front Bioeng Biotechnol. 2024 Feb 8;12:1355009. doi: 10.3389/fbioe.2024.1355009 (PMC10882546; doi:10.3389/fbioe.2024.1355009)
Supplement: Supplementary file 1 [file DataSheet1.PDF]

Decreasing acid value of fatty acid ethyl ester products using complex enzymes

Supplementary Material

1 Supplementary Figures and Tables

1.1 Supplementary Figures

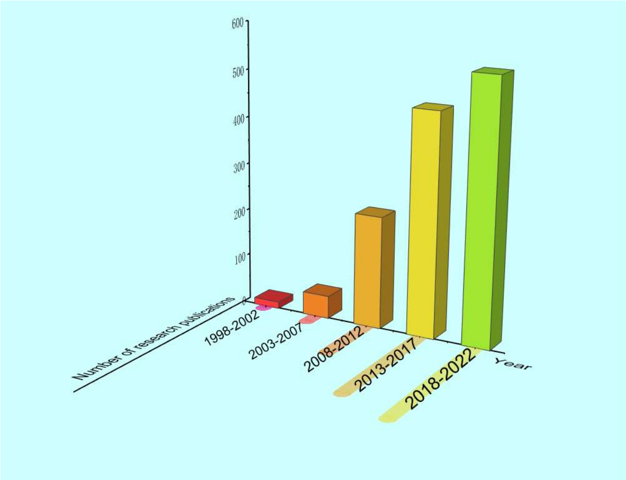

Figure 1. Number of publications per five years on ethanolysis for biodiesel production (data collected from Elsevier ScienceDirect database).

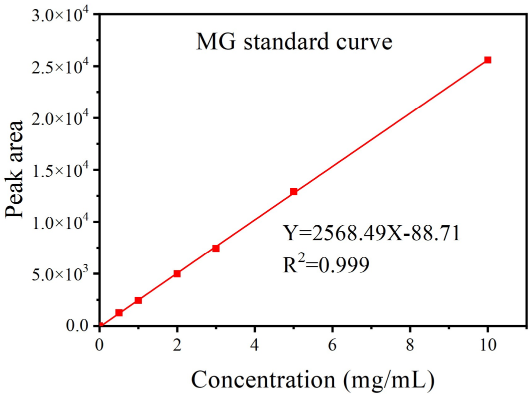

a) Monoglycerides standard curve

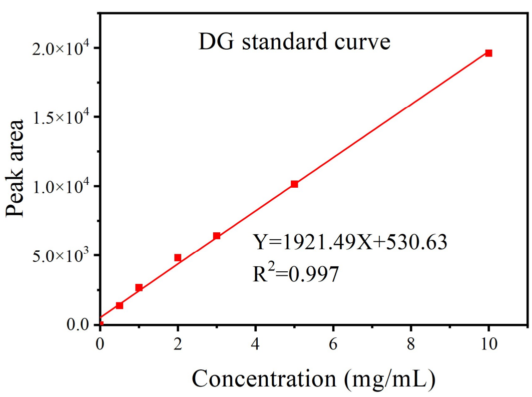

b) Diglycerides standard curve

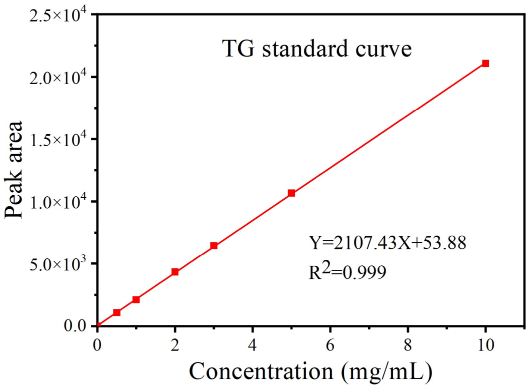

c) Triglycerides standard curve

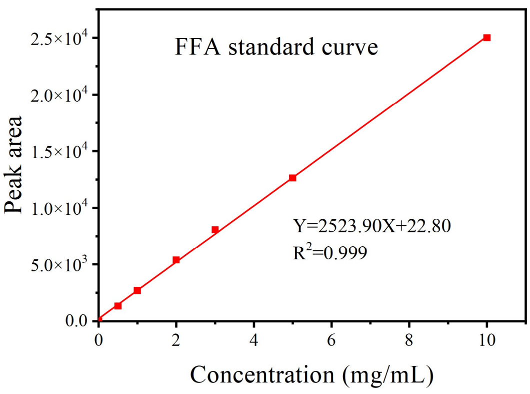

d) Free fatty acid standard curve

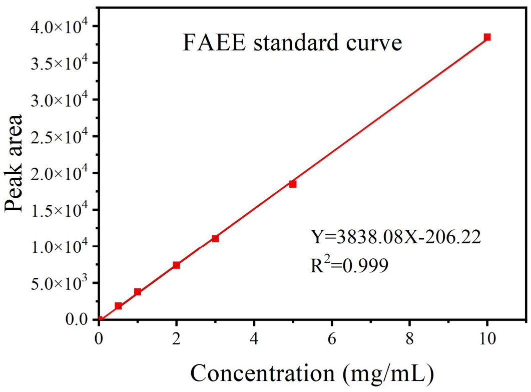

e) FAEE standard curve

Figure 2. Standard curves of each component concentration established with peak area.

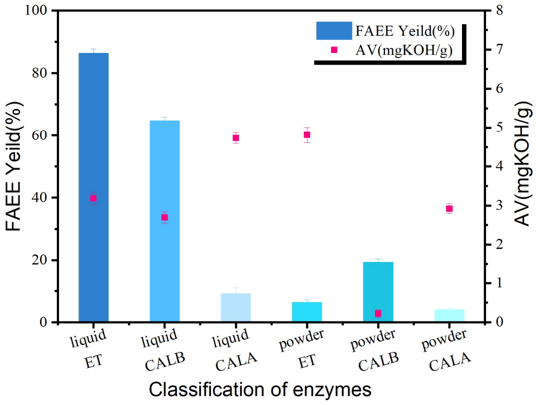

Figure 3. Effect of the type of enzymes on FAEE yield and its acid value. Reaction conditions were 4% liquid lipase load (w/w, SASO), 0.8% of enzyme dry powder (w/w, SASO), 7:1 (mol/mol) substrate

ratio of alcohol to oil, 0.6 g/g of removal agent, 35℃, and 24 h reaction time. The ET labeled in the figure replaces the enzyme Eversa® Transform 2.0.

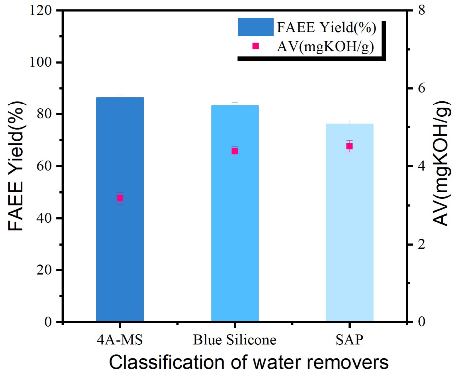

Figure 4. Effect of the type of dehydrating agents on FAEE yield and its acid value. Reaction conditions were 4% addition of liquid lipase Eversa® Transform 2.0(w/w, SASO), alcohol to oil substrate ratio 7:1 (mol/mol), dehydrating agent addition 0.6 g/g, temperature 35℃ and reaction time 24 h.

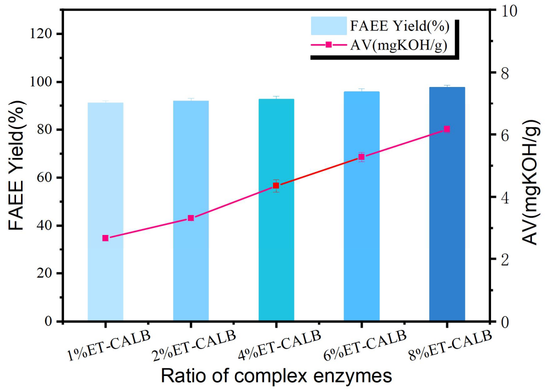

Figure 5. Effect of enzyme compositions on FAEE yield and its acid value. Reaction conditions were alcohol-oil substrate ratio 7:1 (mol/mol), temperature 35℃, reaction time 24 h, and the enzyme dry powder CALB was added at 0.8% (w/w, SASO). The liquid lipase Eversa® Transform 2.0 was added at 1-8% (w/w, SASO).

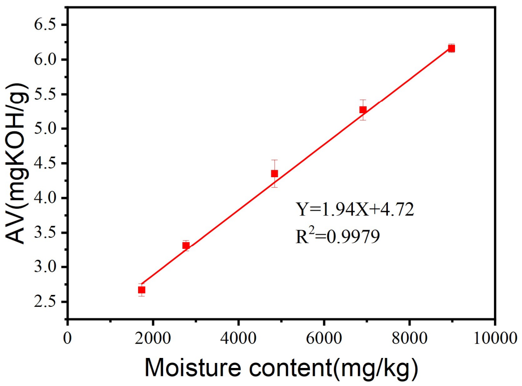

Figure 6. Relationship between moisture content and acid value of FAEE

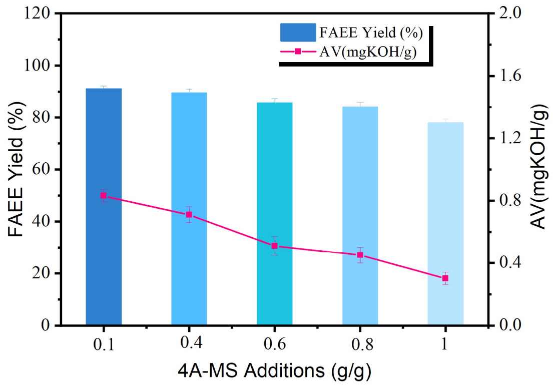

Figure 7. Effect of 4A-MS addition on FAEE yield and its acid value. Reaction conditions were 0.8% addition of enzyme dry powder CALB (w/w, SASO), 1% addition of liquid lipase Eversa® Transform 2.0 (w/w, SASO), alcohol-oil substrate ratio of 7:1 (mol/mol), temperature 35℃ and reaction time 24 h.

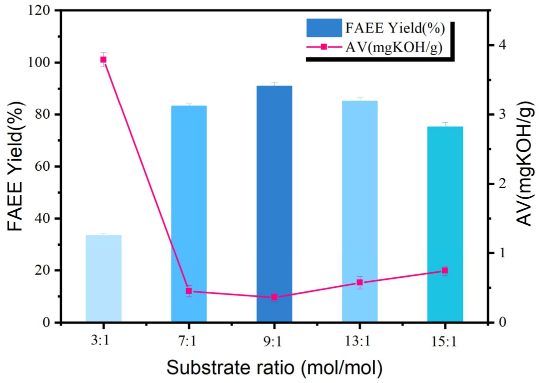

Figure 8. Effect of substrate ratio on FAEE yield and its acid value. Reaction conditions were 1% addition of liquid lipase Eversa® Transform 2.0 and 0.8% enzyme dry powder CALB (w/w, SASO), temperature 35℃, 4A-MS addition load 0.8 g/g and reaction time 24 h.

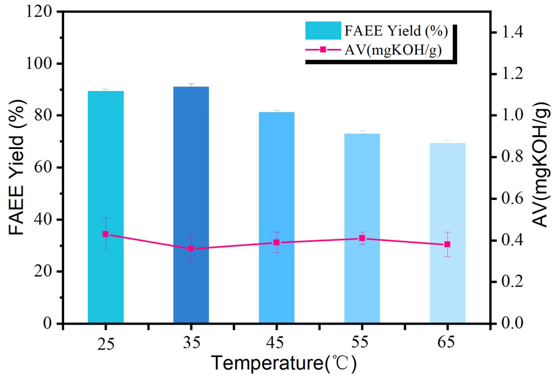

Figure 9. Effect of temperature on FAEE yield and its acid value. Reaction conditions were 0.8% addition of enzyme dry powder CALB and 1% liquid lipase Eversa® Transform 2.0 (w/w, SASO), alcohol-oil substrate ratio 9:1 (mol/mol), 4A-MS addition load 0.8 g/g, and reaction time 24 h.

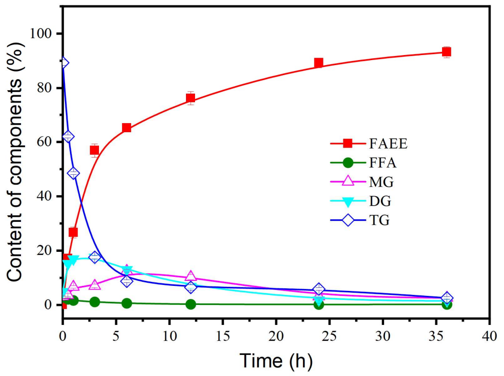

Figure 10. Variation of the content of each component with time

1.2 Supplementary Tables

Table 1. Physico-chemical index of fatty acid ethyl ester of SASO

Physico-chemical indexes	SASO FAEE	ASTM D6751
Ethyl ester content (%, w/w)	97.6	≥ 96.5
Acid value (mgKOH/g)	0.42	≤ 0.5
Moisture content (%)	0.046	-
Kinematic viscosity (40°C, mm^2/s)	4.54	1.9~6.0
Flash point (°C)	174	≥ 93
Densities (15°C, g/cm^3)	0.87	-
Oxidative stability (110°C, h)	0.56	≥ 3
Iodine value (g/100g)	126.47	-
Cetane number	49.09	≥ 47
Copper sheet corrosion (50°C, 3 h)	1	≤ 1
